# Initial Radiological Misdiagnosis of Choledochal Cyst in Pregnancy Leading to Unnecessary Laparotomy: The Approach to Abdomino-Pelvic Cyst and Management

**DOI:** 10.7759/cureus.94963

**Published:** 2025-10-20

**Authors:** Saqib Ahmed, Karthik Raghunath, Joish Upendra

**Affiliations:** 1 Medicine and Surgery, SDM College of Medical Sciences and Hospital, Dharwad, IND; 2 Radiology, SDM College of Medical Sciences and Hospital, Dharwad, IND

**Keywords:** case report, choledochal cyst, diagnostic imaging, laparotomy, misdiagnosis, mri, percutaneous aspiration, pregnancy, ultrasound, usg

## Abstract

A 20-year-old primary gravida at 26 weeks of gestation presented with acute abdominal pain. At a rural facility that she had consulted earlier, abdominal pelvic ultrasonography had suggested adnexal pathology, most likely ovarian torsion, and an exploratory laparotomy had been performed. No adnexal pathology had been found, and the procedure had been abandoned. The patient was transferred to our center in hemodynamic instability. Repeat ultrasonography identified a large right subhepatic cystic lesion with differentials including choledochal cyst (CC), benign retroperitoneal cyst, and ovarian/paraovarian cyst. MRIs subsequently confirmed a giant right upper quadrant cyst compressing hepatobiliary structures, correlating with the development of clinical jaundice. This clarified the etiology and eliminated the need for further unnecessary gynecologic surgery.

The patient was managed with ultrasound-guided percutaneous drainage, which safely decompressed the biliary system, relieved mass effect, and reduced rupture risk in the context of high maternal-fetal surgical risk. The intervention stabilized the patient, facilitated definitive cyst excision before normal vaginal delivery at 36+6 weeks. Based on this experience and a review of the relevant literature, we suggest that in selected cases of undifferentiated abdomino-pelvic cysts during pregnancy, particularly in resource-limited or emergency settings without access to advanced imaging, ultrasound-guided percutaneous drainage may be a valuable diagnostic and temporizing measure. This approach can safely bridge patients to definitive management and may help avoid unnecessary laparotomy.

## Introduction

Choledochal cysts (CCs) are rare congenital dilatations of the biliary tree and represent the second most common biliary duct anomaly after biliary atresia. They occur three to four times more frequently in females than in males. The incidence ranges from approximately one in 100,000 to 150,000 live births in Western populations, but can be as high as one in 1,000 in certain regions of Asia [2,3]. While most CCs are identified in childhood, approximately 20-25% are not diagnosed until adulthood. The Todani classification system is the most commonly used, with Type I cysts accounting for around 90% of cases. Maternal CCs represent a much rarer clinical entity. A recent systematic review by Augustin et al. identified only 97 reported cases, of which 88 were diagnosed during pregnancy [1].

The presenting symptoms are often nonspecific - abdominal pain occurs in approximately 81% of cases, and jaundice in about 60% -which can overlap with common obstetric, gynecologic, or general surgical conditions, particularly in the context of pregnancy [1]. Pregnancy distorts abdominal anatomy due to gravid uterine enlargement and can mask hepatobiliary pathology. Large CCs in pregnancy can mimic other cystic masses; e.g., a right upper quadrant cyst might be misinterpreted as an adnexal lesion, such as in our case. This leads to unnecessary, difficult, and dangerous interventions that pose significant risk. Laparotomy during pregnancy carries notable maternal-fetal risks, including higher rates of preterm birth, low birth weight, gestational hypertension, preterm labor, and cesarean delivery [5].

The diagnosis of a large abdomino-pelvic cystic mass can be challenging, and may involve a broad differential diagnosis, which includes ovarian cysts, hepatic cysts, and mesenteric cysts, among others [6]. Imaging plays a key role in addressing this challenge. Ultrasonography (USG) is the first-line modality in pregnancy, but its utility is often limited due to an enlarged uterus and poor delineation of the biliary tree, making it challenging for even experienced sonographers [6,7]. The next step involves the use of MRI/MR cholangiopancreatography (MRCP). It can trace the cyst to the extrahepatic bile duct and reveal proximal biliary dilatation, which is indicative of a CC [6]. However, this imaging modality is not always readily available and, due to its high cost, is not commonly utilized [8], particularly in India [9]. Hence, accurate diagnosis becomes even more critical to avoid unnecessary surgical interventions and to ensure appropriate and timely perinatal management.

## Case presentation

A 20-year-old primigravida at 26 weeks of gestation initially presented to an external rural medical facility in Koppal (RMFK) on December 27, 2023, with a four-day history of worsening abdominal pain and vomiting, which were exacerbated by food intake. Based on the reports provided by the patient, the abdominal pelvic USG conducted at RMFK indicated the presence of an adnexal mass, most likely ovarian torsion. However, no imaging files or Doppler study results were made available.. She underwent emergency exploratory laparotomy on the same day for suspected ovarian torsion. Intraoperative findings (as per RMFK report) included normal ovaries and a cyst appearing to be of subhepatic origin. The abdomen was then closed without any intraoperative intervention. We suspect that the combination of an inconclusive USG finding and clinical features had prompted the initial misdiagnosis. 

The patient later developed postoperative complications, including breathlessness and tachycardia during transfusion. On December 28, she was transferred to SDM Hospital in Dharwad, where she presented with unstable vital signs. On examination, she was conscious, oriented, but tachypneic and tachycardic. Her vitals were as follows: heart rate (HR): 112 bpm; BP: 130/90 mmHg | respiratory rate (RR): 22 bpm; and SpO_2_: 86%. The abdomen was distended, featuring a central umbilicus and linea nigra, with hernial orifices intact. No dilated veins or striae were observed.

**Figure 1:**
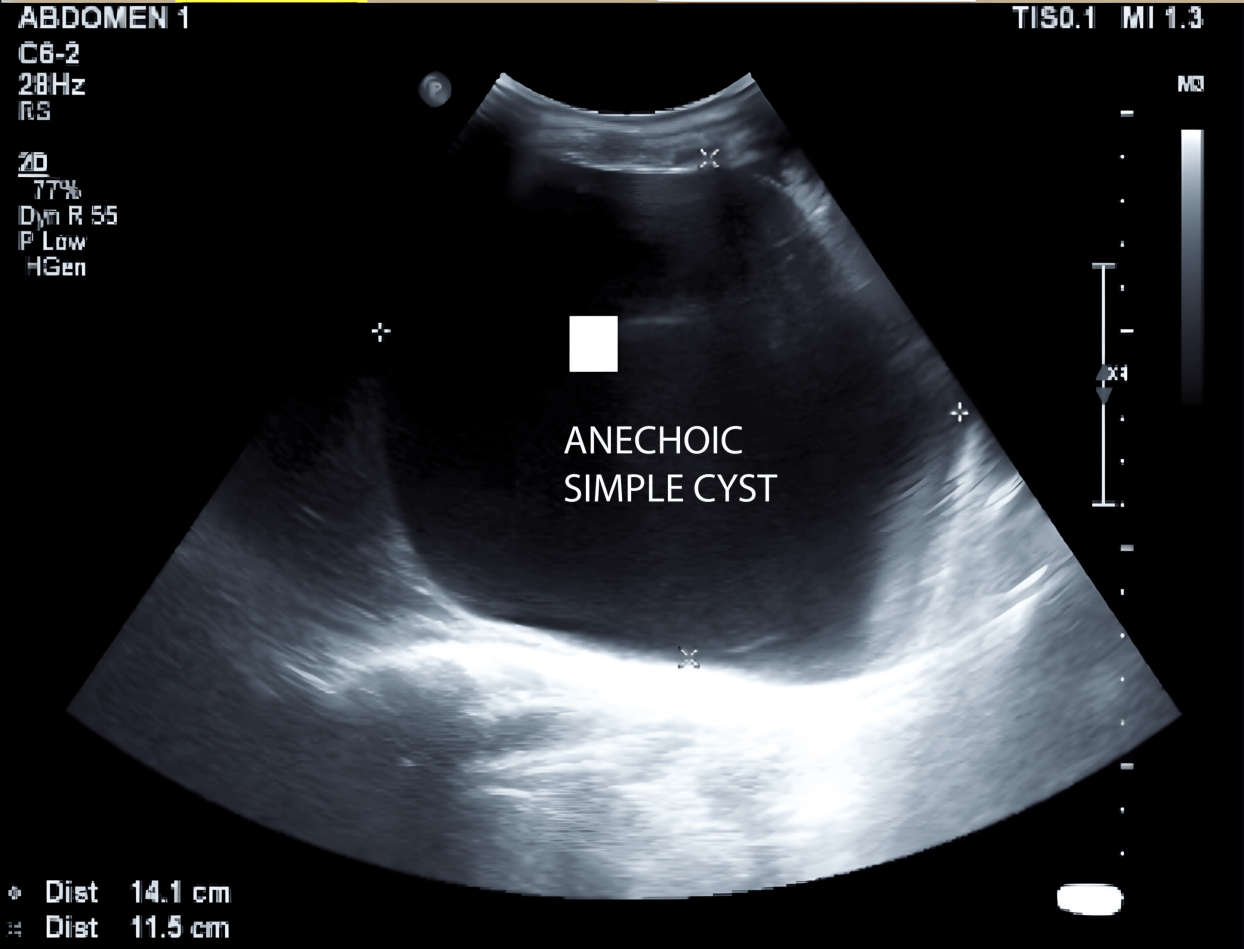
Ultrasound of the abdomen - image 1 A large anechoic cyst measuring 11.5 x 14 cm is seen in the right hepatic region. It has thin walls with no evidence of septations, solid components, or calcifications within

**Figure 2:**
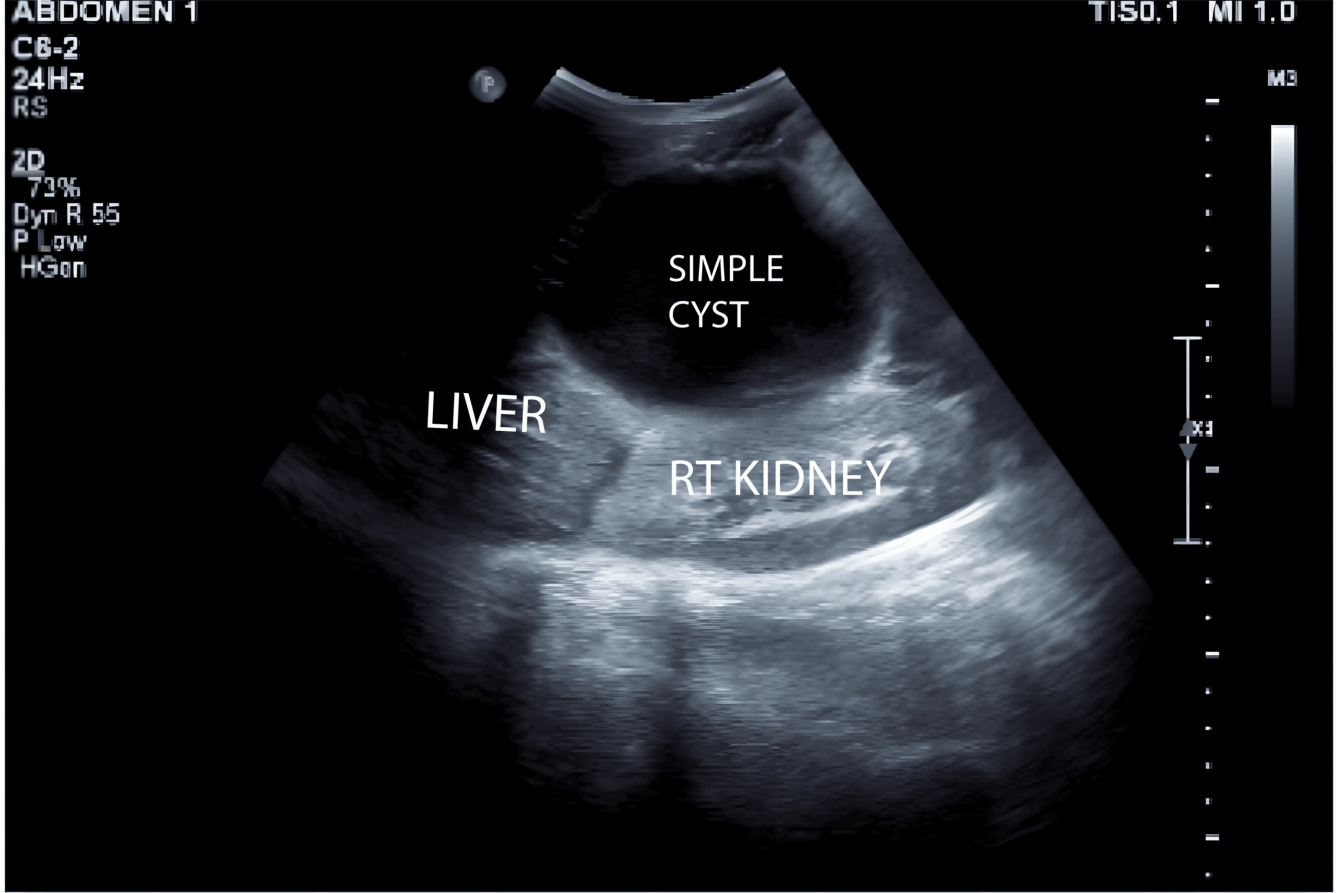
Ultrasound of the abdomen - image 2 The image shows a simple anechoic cyst indenting the right lobe of the liver (superiorly) and compressing the right kidney (posteriorly)

**Figure 3:**
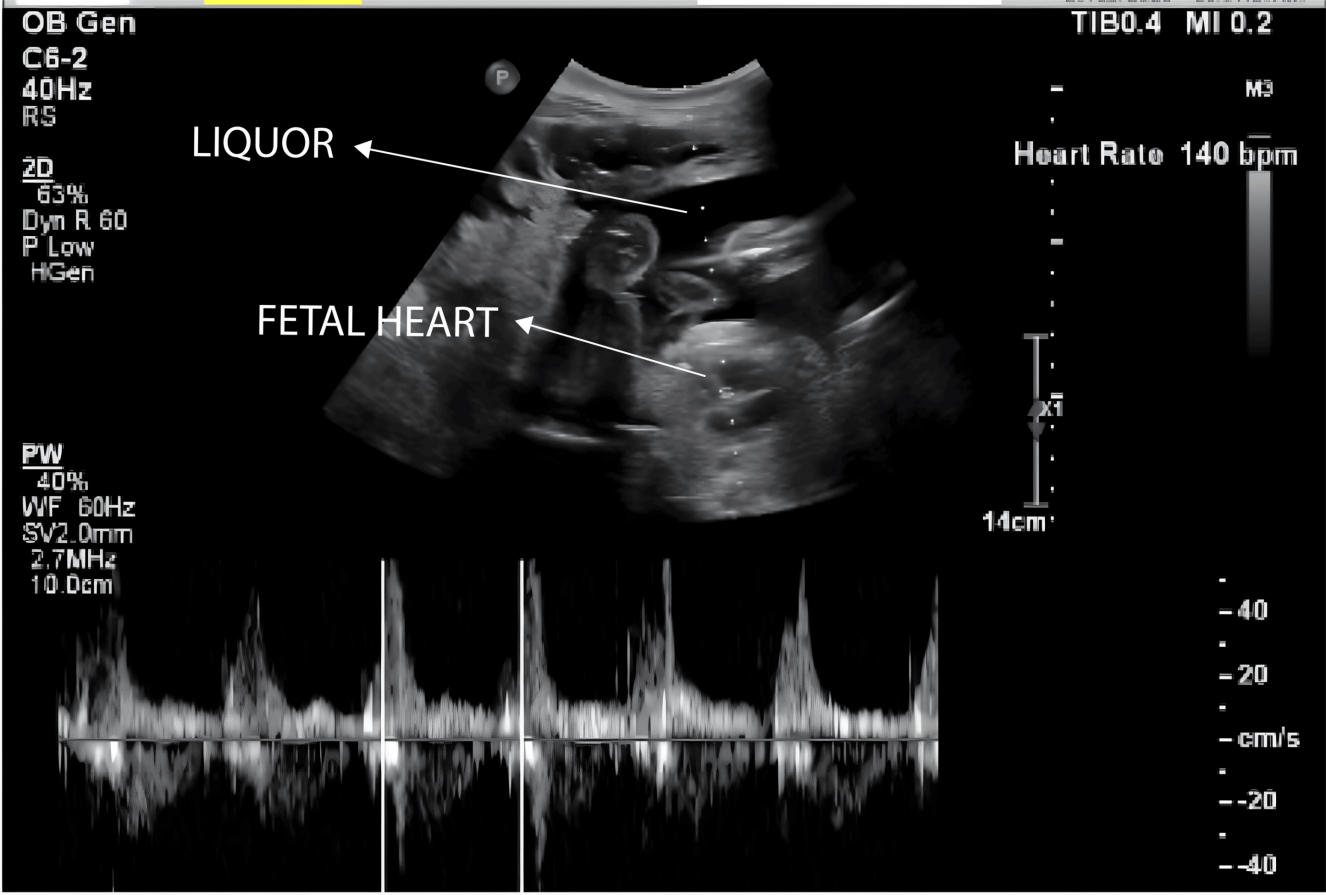
Ultrasound of the abdomen and fetal Doppler of the uterus The image revealed a single live intrauterine gestation (SLIUG) of 26 weeks

The uterus was consistent with a 28-week gestation. Dressing from prior laparotomy was intact with minimal soakage. Midline and Pfannenstiel sutures were noted, and there was diffuse abdominal tenderness with guarding but no rigidity. There was no localized increase in temperature. Percussion revealed a tympanic note. Bowel sounds and fetal heart sounds were present. Per speculum examination, the cervix and vagina appeared healthy. On vaginal examination, the cervix was uneffaced, admitting one finger, with the vertex at -3 station. Respiratory examination revealed bilateral crepitus. Cardiovascular assessment indicated normal S1 and S2 heart sounds with no murmurs. The patient had no clinical jaundice or pruritus. Baseline blood tests were performed, and liver function tests (LFTs) were within normal limits. An abdomino-pelvic USG (29/12/23) then revealed a large anechoic cystic lesion measuring 11.5 x 14.0 cm in the right subhepatic region (Figure 1) with thin walls, without septations, solid components, or calcifications. The lesion indented the right liver lobe superiorly and compressed the right kidney posteriorly. Communication to the origin could not be ascertained (Figure 2). Differentials at this time included CC, retroperitoneal cyst, and ovarian/paraovarian cyst. Uterus showed a single live intrauterine gestation at 26 weeks (Figure 3).

**Figure 4:**
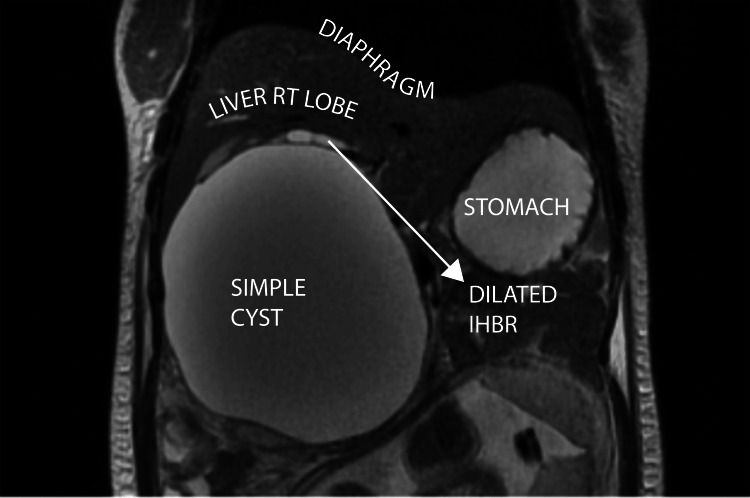
Coronal T2-weighted MRI - image 1 The image shows a T2 hyperintense cystic lesion measuring 14.2 x 12 x 12.8 cm. It is indenting the right lobe of the liver (superiorly) MRI: magnetic resonance imaging

**Figure 5:**
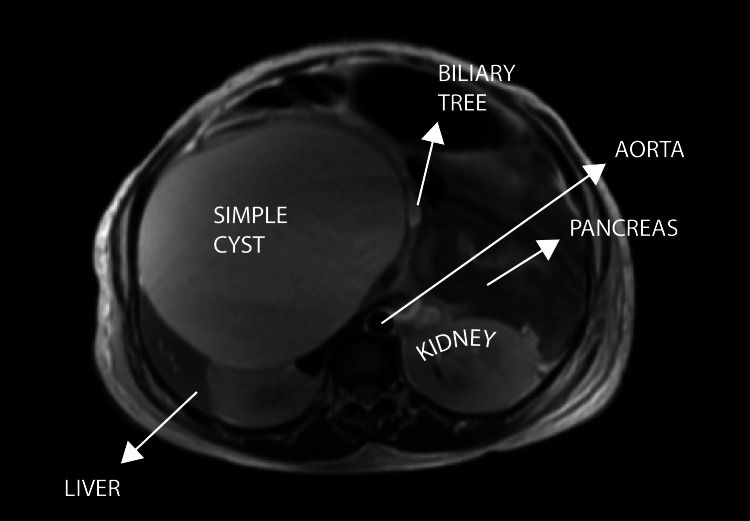
Axial T2-weighted MRI The image shows a cyst compressing the right kidney (inferiorly) and indenting and displacing the head of the pancreas (medially) MRI: magnetic resonance imaging

MRI (29/12/23) at 26 weeks of gestation confirmed a large, well-defined T2 hyperintense cystic lesion measuring 14.2 x 12.0 x 12.8 cm in the right subhepatic area, superiorly indenting the right lobe of the liver (Figure 4). The lesion was compressing the right kidney inferiorly and displacing the ancreatic head medially (Figure 5).

**Figure 6:**
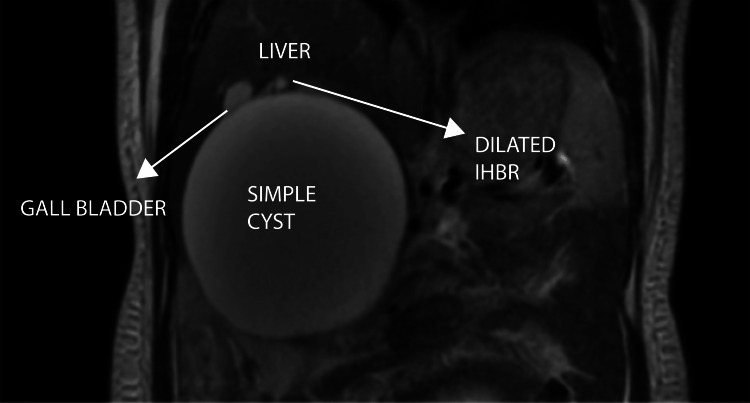
Coronal T2-weighted MRI - image 2 The image shows the lesion compressing the proximal biliary tract with mild central IHBRD. CHD and CBD could not be traced separately from the lesion MRI: magnetic resonance imaging; IHBRD: intrahepatic biliary radicle dilatation; CHD: common hepatic duct; CBD: common bile duct

It caused proximal biliary tract compression with mild central intrahepatic biliary radicle dilatation (IHBRD). The common hepatic duct (CHD) and common bile duct (CBD) could not be traced separately from the lesion (Figure 6).

**Figure 7:**
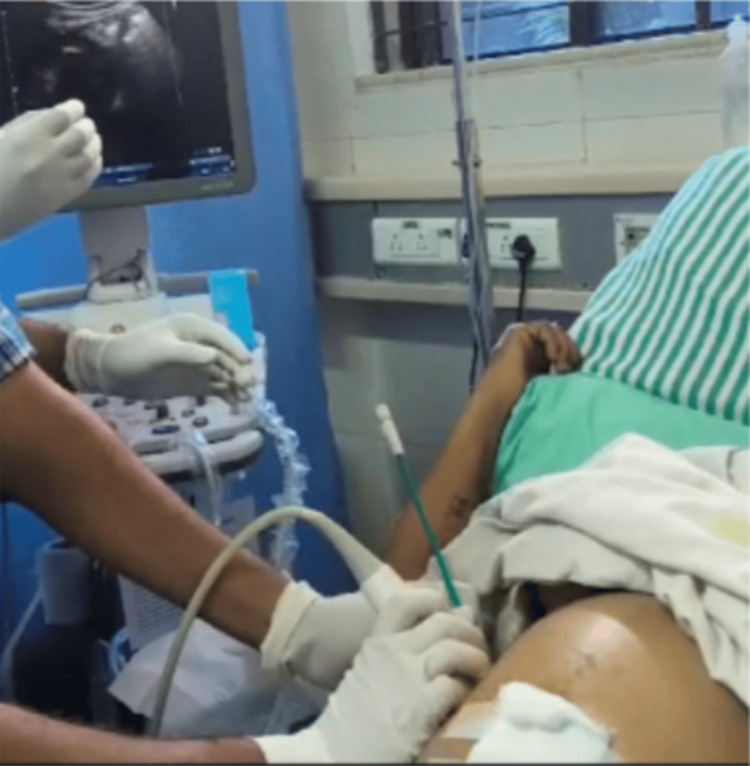
Ultrasound-guided percutaneous aspiration with pigtail placement - image 1

**Figure 8:**
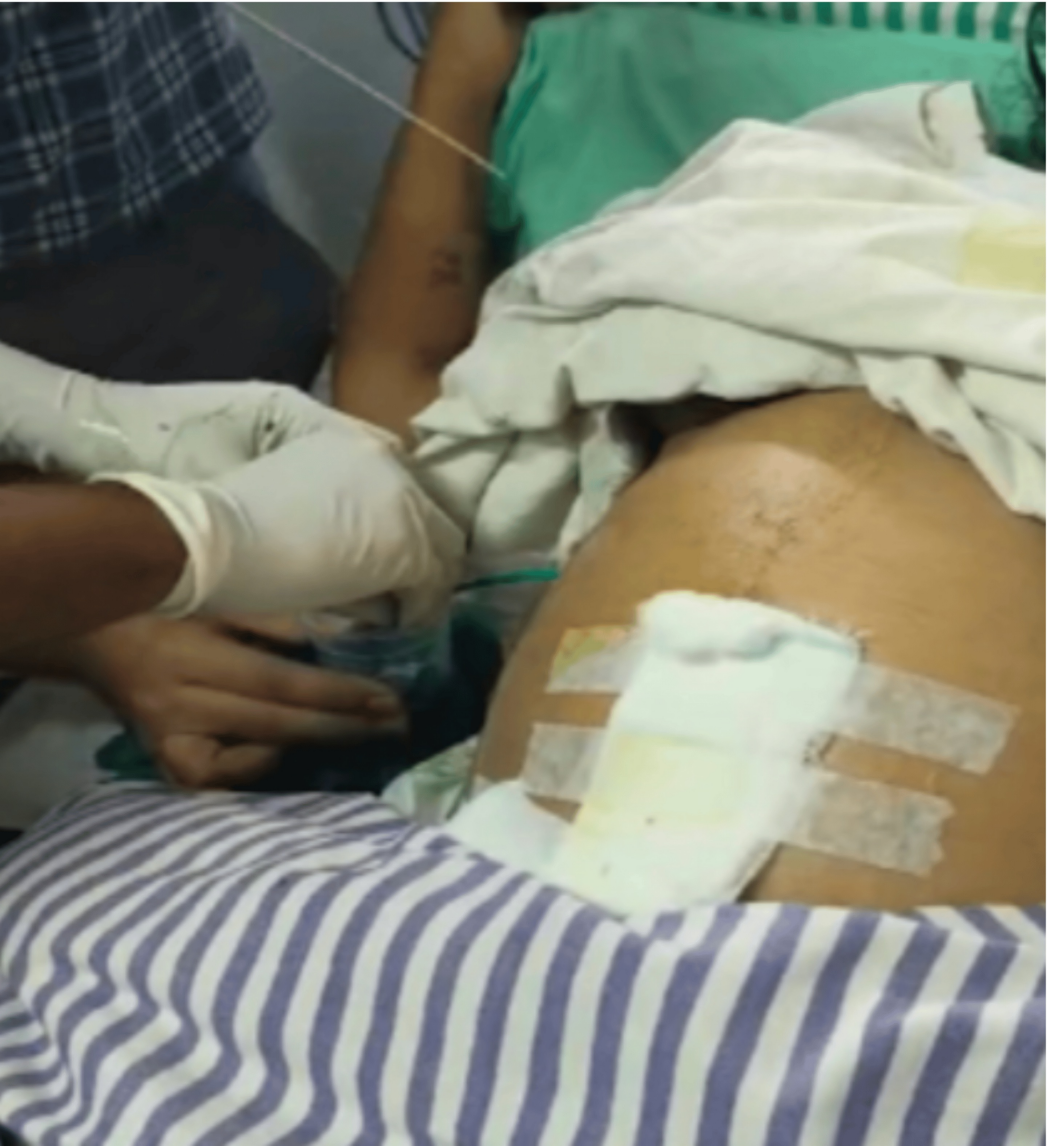
Ultrasound-guided percutaneous aspiration with pigtail placement - image 2 The image shows dressing over the midline and Pfannensteil sutures from a laparotomy performed outside at RMFK RMFK: Rural Medical Facility, Koppal

The patient developed clinical icterus on day two of admission (29/12), along with persistent low sats (86%) and respiratory distress. Labs on day four (31/12) revealed markedly raised total serum bilirubin (TSB), direct bilirubin (DB), alkaline phosphatase (ALP), and gamma-glutamyl transpeptidase (GGT) values, and her respiratory functions deteriorated. Percutaneous catheter drainage (PCD) under USG was planned to decompress the cyst and relieve respiratory distress. Percutaneous aspiration with pigtail placement was performed under strict aseptic precautions (2/1/23) (Figures 7, 8). Aspiration yielded approximately 1100 ml of grossly bilious fluid. Fetal monitoring was done under OBGYN guidance.

**Figure 9:**
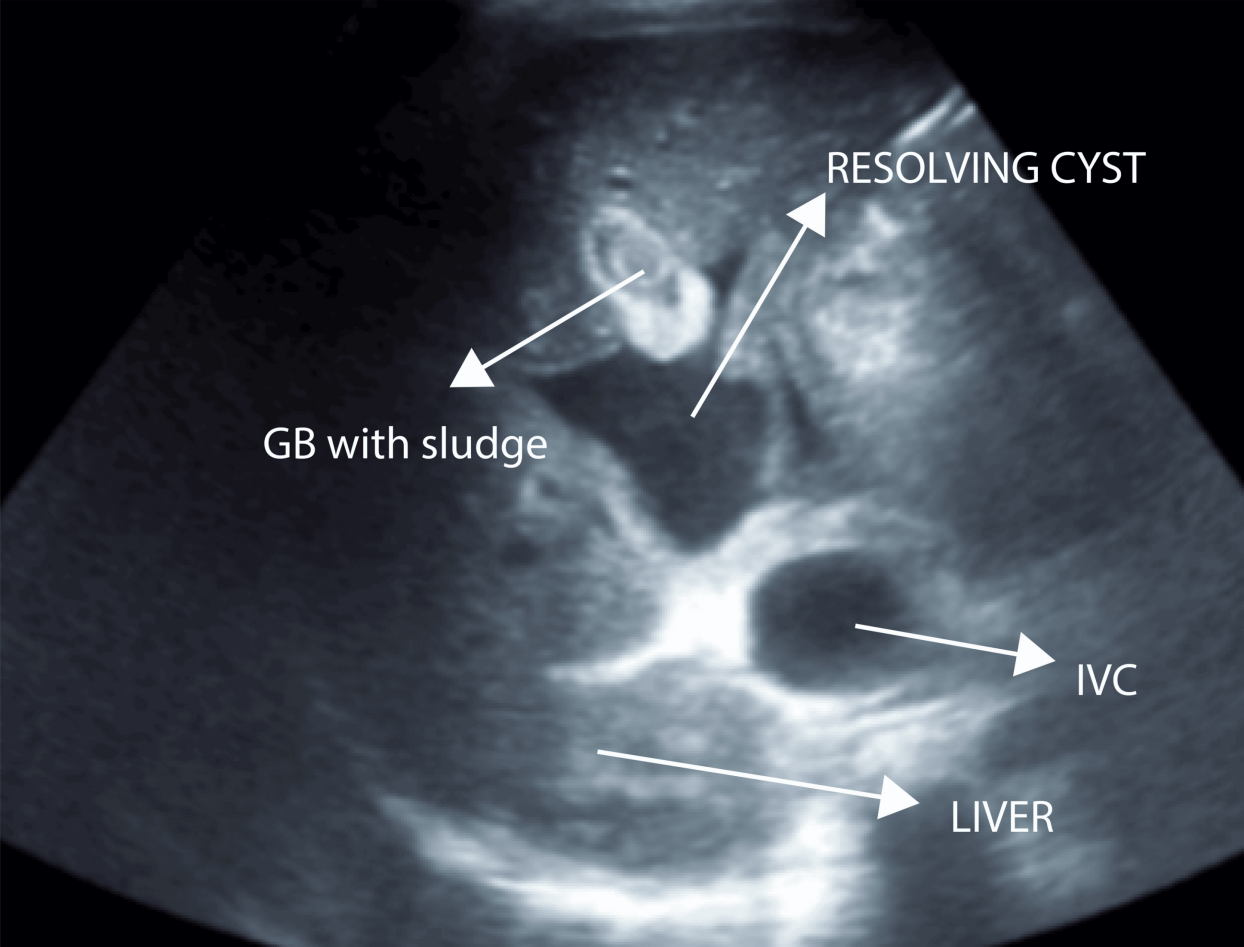
Post-procedure ultrasound of the abdomen - image 1 Resolution of the cystic lesion is noted, now measuring 4.5 x 4 x 2.5 cm. GB is partially distended and shows sludge within.CBD could not be traced, and no IHBR dilatation was seen GB: gall bladder; CBD: common bile duct; IHBR: intrahepatic biliary dilatation

**Figure 10:**
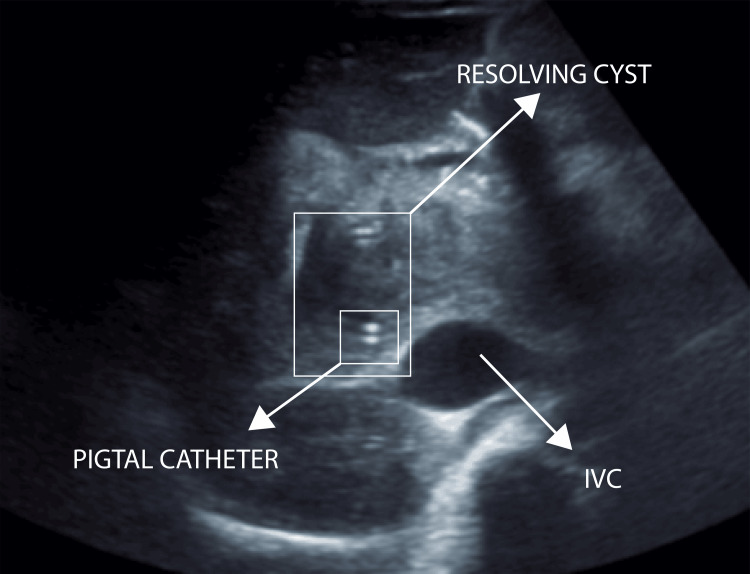
Post-procedure ultrasound of the abdomen - image 2 The image shows the resolved cyst with a pigtail catheter in situ

Post-procedure USG imaging showed significant resolution of the cyst with residual lesion measuring 4.5 x 4.0 x 2.5 cm (20-25 cc) with pigtail in situ anterior to the portal vein adjacent to the gallbladder. The gallbladder was partially distended with sludge. CBD could not be traced, and no IHBRD was seen (Figures 9, 10).

Daily serial monitoring of the pigtail drain output (billious fluid) was undertaken (600 ml, 400 ml, 350 ml). Our differential diagnosis at this point included biloma and CC Type 1A. A provisional diagnosis of CC Type 1A was more favorable, as persistent draining fluid over the following days suggested a communication with the biliary tree. Additionally, since the cyst could not be clearly separated from both the CHD and CBD, Type 1A was the most fitting diagnosis at this stage. Fetal status was assessed regularly under the guidance of OBGYN, with daily reviews and recommendations followed. Aseptic surgical site dressing was performed daily. On post-procedure day 1 (PPD-1), icterus was reduced. The patient had improved oxygen saturation levels and reduced respiratory distress.

**Figure 11:**
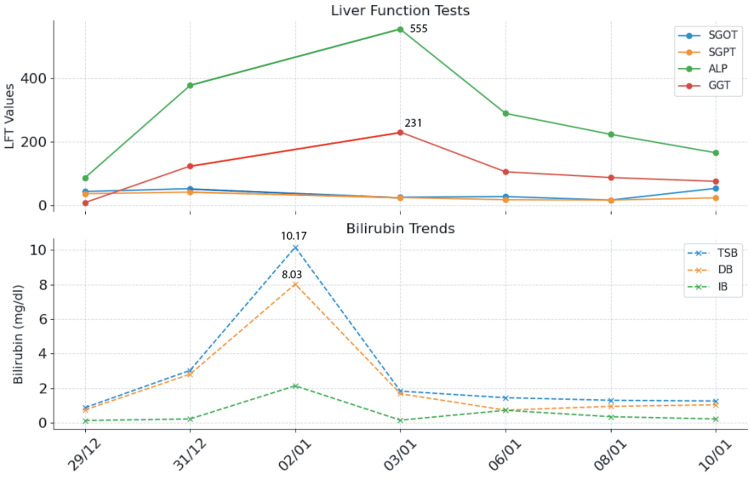
Trends in laboratory analysis (liver enzymes and serum bilirubin trends) TSB: total serum bilirubin; DB: direct bilirubin; IB: indirect bilirubin; SGOT: serum glutamic-oxaloacetic transaminase; SGPT: serum glutamic-pyruvic transaminase; ALP: alkaline phosphatase; GGT: gamma-glutamyl transferase

For definitive management, the patient was planned for surgery after a thorough multidisciplinary team discussion. She underwent exploratory laparotomy + excision of cyst + hepaticojejunostomy + jejunojejunostomy (9/1/24) under general anaesthesia + epidural anaesthesia (Table 1), seven days after the PCD insertion. Intraoperative exploration revealed communication of the cyst with the biliary tree, continuous with the CBD, confirming the presence of CC Type 1C (Table 1). She tolerated the procedure well. Serial monitoring of the drain (subhepatic and subcutaneous) output was done and found to be non-bilious, serosanguinous in nature. Subsequent histopathology of the cyst wall confirmed CC with spongioid hyperplasia of the gall bladder (Table 1). Fifty-eight days later, at 36+6 weeks of gestation, she underwent an uncomplicated spontaneous vaginal delivery (7/3/24), under medical supervision, with no intrapartum complications for either mother or neonate. Subsequent maternal and neonatal course after delivery could not be ascertained as the patient was lost to follow-up. Table 2 presents the serial liver panel findings from admission to surgery. Figure 11 depicts the trends in laboratory analysis (liver enzymes and serum bilirubin trends).

**Table 1:** Imaging, surgical, and biopsy findings USG: ultrasound; RMFK: rural medical facility, Koppal; SDM: SDM Hospital; DDx: differential diagnosis; SLIUG: single live intrauterine gestation; MRI: magnetic resonance imaging; AP: anteroposterior; ML: mediolateral; CC: cephalocaudal; IHBRD: intrahepatic biliary radicle dilatation; CHD: common hepatic duct; CBD: common bile duct; GB: gall bladder; PCD: percutaneous catheter drainage

Date	Modality	Findings
27/12/23	USG abdomino-pelvic (RMFK)	RMF transfer note; detailed findings and Doppler assessment not provided. Gravid uterus corresponding to 26 weeks of gestation with SLIUG. Adjacent to the uterus, a large, anechoic, thin-walled simple cyst was observed. Ovaries were not confidently visualized separately from the cystic mass. Impression: right ovarian cyst - adnexal torsion
27/12/23	Intra-op laparotomy (RMFK)	RMF transfer note. Gravid uterus corresponding to 26 weeks of gestation. Large tense cystic mass in the RUQ extending below the umbilicus and displacing bowel loops. The cyst appears to be of subhepatic origin, with no obvious communication to the uterus or adnexal structures observed intraoperatively. Mild ascites present. No evidence of torsion noted
29/12/23	USG abdomino-pelvic (SDM)	A large anechoic cystic lesion measuring 11.5 x 14 cm is seen in the right subhepatic region. It has thin walls with no evidence of septations/solid components/calcifications within. Superiorly indenting the right lobe of the liver, posteriorly compressing the right kidney. Ovaries not visualized. The uterus shows SLIUG with gestation of 26 weeks. DDx: choledochal cyst, retroperitoneal lymphatic cyst, ovarian/paraovarian cyst
29/12/23	MRI abdomen (SDM)	A large, well-defined T2 hyperintense cystic lesion seen in the right subhepatic region measuring 14.2 x 12 x 12.8 cm (AP x ML x CC). Superiorly, the lesion is indenting the right lobe of the liver. Inferiorly, it is compressing over the right kidney. Medially, it is indenting and displacing the head of the pancreas. The lesion is causing compression over the proximal biliary tract with mild central IHBRD. CHD and CBD could not be traced separately from the lesion. DDx: choledochal cyst (most likely), retroperitoneal lymphatic cyst, non-pancreatic pseudocyst
2/1/23	USG post-PCD (SDM)	Significant resolution of cystic lesion noted, now measuring 4.5 x 4.0 x 2.5 cm (20-25 cc) with pigtail in situ anterior to the portal vein adjacent to the GB. GB is partially distended and shows sludge within. CBD could not be traced. No IHBRD seen. DDx: choledochal cyst Type 1A, biloma (unlikely)
9/1/24	Intra-op definitive excision surgery (SDM)	Choledochal cyst measuring 14 x 12 cm. GB is directly draining into the cyst. Short and thin common hepatic duct noted. Multiple adhesions are noted between the cyst wall and surrounding structures. Confirmatory diagnosis: choledochal cyst Type 1C
10/1/24	Histopathological examination (SDM)	Focal columnar epithelial lining with subepithelial fibrocollagenous tissue with sparse lymphocytic infiltration and few dilated and congested blood vessels. GB sections show partially ulcerated mucosa with mucosal gland hyperplasia. Transmural mixed inflammatory cell infiltrate comprising lymphocytes and neutrophils is noted. Rokitansky-Aschoff sinuses are seen. Diagnosis of specimen: biopsy of choledochal cyst wall - choledochal cyst with spongioid hyperplasia of GB

**Table 2:** Serial liver panel from admission to surgery PCD: percutaneous catheter drainage; TSB: total serum bilirubin; DB: direct bilirubin; IB: indirect bilirubin; SGOT: serum glutamic-oxaloacetic transaminase; SGPT: serum glutamic-pyruvic transaminase; ALP: alkaline phosphatase; GGT: gamma-glutamyl transferase; TP: total protein

Parameter	29/12	31/12	02/01 (PCD)	03/01	6/1	8/1	10/1 (surgery)
TSB, mg/dL	0.87	3.03	10.17	1.83	1.45	1.3	1.26
DB, mg/dL	0.74	2.81	8.03	1.68	0.73	0.95	1.04
IB, mg/dL	0.13	0.22	2.14	0.15	0.72	0.35	0.22
SGOT, U/L	44	53		26	28	17	54
SGPT, U/L	37	42		25	18	17	24
ALP, U/L	87	378		555	290	224	166
GGT, U/L	9	124		231	106	88	76
TP, g/dL	4.6	5		3.8	4.5	5.1	4.4

## Discussion

Our patient’s initial presentation with acute abdominal pain at approximately 28 weeks of gestation aligns with the typical pattern observed in most reported cases. Notably, she lacked symptoms such as jaundice and pruritus, which are present in about 60% and 44% of CC cases diagnosed during pregnancy between 24 and 36 weeks [1]. Females are predominantly affected, necessitating careful differentiation from obstetric-gynecological conditions, especially during pregnancy [10,11]. Despite its rarity, the clinical implications and potential complications of CC, including cholangitis, pancreatitis, and later malignant transformation, highlight the importance of accurate and timely diagnosis [12].

Ultrasound is highly sensitive to fluid-filled structures and remains a useful screening tool. CCs can present with specific clues and findings such as tubular connection of the cyst to the dilated bile ducts or the presence of biliary sludge/stones within the cyst [4]. Initial abdominopelvic USG imaging at our center confirmed the presence of a large abdominal cyst with internal mobile echoes appearing consistent with biliary sludge. The cyst measured approximately 12-14 cm in diameter, and its large size, combined with the presence of the gravid uterus, made it difficult to clearly identify its point of origin. The pancreas could not be clearly visualized either. Koh et al. have stressed that when a large cystic mass is seen in the upper abdomen of a pregnant patient, a CC must be included in the differential [7].

No obvious uterine abnormalities were seen; however, the ovaries were not visualized on this scan, likely due to their displacement higher in the pelvis from the upward shift of the gravid uterus, which limited visibility. Although the cyst was located in the right upper quadrant (RUQ), its anechoic appearance, uncertain origin, and positional changes led us to consider an ovarian or paraovarian cyst as the last differential, after CC and retroperitoneal cyst (Table 1). These nonspecific USG findings reflect those in prior reports where a choledochal cyst in late pregnancy was seen as a large anechoic mass in the RUQ without clear continuity to the biliary tract [8]. 

With a likely suspicion of a CC high on our list, we proceeded with an MRI. MRI is the most strongly recommended imaging modality of choice for newly diagnosed CCs in pregnancy [1]. Our patient’s MRI (gadolinium contrast was avoided) clearly depicted the cyst’s relationships and impact on adjacent organs (Table 1). The patient’s clinical picture fit the imaging finding of diaphragmatic compression and subsequent respiratory compromise, along with IVC and renal vein compression being the likely contributors to ascites and venous congestion. These extensive mass effects usually correspond to a truly “giant” cyst. The mean size of CCs in pregnancy is ~12.5 cm, and cases over 15 cm are infrequent. There are rare documentations of cysts up to 20-40 cms, which have a high likelihood of sudden rupture [1]. 

ERCP, though capable of direct visualization and drainage via stenting, is sparingly used in pregnancy due to fluoroscopy and sedation risks. In a similar case, ERCP failed since the enlarged uterus and cyst distorted duodenal anatomy [7]. Given our MRI findings, ERCP was deemed unnecessary before intervention. To improve clarity, Morales-Maza et al. recommend post-partum MRI imaging for visualizing the cyst architecture. Even so, waiting until after delivery can complicate the patient's condition in critical situations such as ours [4]. Advanced imaging techniques, such as MRI and MRCP, remain crucial for the precise identification and management of CC, with MRCP being the gold standard given its high sensitivity and specificity [13]. Nonetheless, like other large abdomino-pelvic presentations in pregnancy, establishing a confirmatory diagnosis can still be challenging without invasive measures.

Differential diagnosis and challenges of a large abdomino-pelvic cyst

Due to the incomplete imaging information provided by RMFK, we conducted a literature review to explore potential factors that may have contributed to the initial misdiagnosis and the subsequent laparotomy. Notably, the misdiagnosis of ovarian torsion by RMFK is not unprecedented.. Fok et al. have described one case where a CC was misinterpreted as an ovarian pathology on prenatal ultrasound during two consecutive pregnancies. The misinterpretation was only realized postpartum when the patient developed cholangitis [14]. While ovarian cysts often present as large cystic masses during pregnancy, other potential causes should also be taken into consideration.

Table 3 provides a summary of case reports involving large abdomino-pelvic cyst types encountered in pregnancy (excluding CCs) that presented with similar features, including USG appearance, clinical features, and diagnostic challenges. We noted that all of them exhibited unavailable or very broad working diagnoses during imaging, and challenges were encountered for subsequent management, especially with acute presentations. Due to the more common incidence of ovarian pathologies on imaging, we observed many non-ovarian differentials being overlooked or considered second or third line, as in the discussed cases [15-19]. Although the list of differentials is not exhaustive, the patterns of vague complaints, inconclusive clinical findings, and nonspecific USG findings call for a broader approach.

**Table 3:** Analysis of differential diagnoses of large abdomino-pelvic cysts (excluding CC) from cases with similar presentations encountered in pregnancy The cases illustrate the difficulty of diagnosis (using USG and MRI) when the cysts are large, and in some cases, the difficult management plans they lead to w.o.g: weeks of gestation; h/o: history of; USG: ultrasound; DDx: differential diagnosis; PIC: peritoneal inclusion cyst; f/b: followed by; Pt: patient; HPE: histopathological exam; PDx: provisional diagnosis

Cyst type	Clinical presentation	USG features (abdomino-pelvic)	MRI (abdomino-pelvic)	Intra-op findings	HPE/definitive diagnosis
Peritoneal inclusion cyst (Hitzerd et al.) [15]	27 yrs/14 w.o.g. Incidental finding on routine USG. No h/o complaints	Findings: multilocular, homogenous, and partially dense cysts posterior to the uterus, measuring 9.5 × 5.5 cm in diameter, DDx: functional ovarian cyst	Findings: large multicystic lesion (with thin septations) located posterior and to the left of the uterus measuring 10 cm in diameter. No free fluid noticed. DDx: presumed origin - ovary ->? ovarian tumor (benign, borderline, malignant)? Non-ovarian tumor (omental cyst, PIC)	Findings: multicystic lesions - translucent and haemorrhagic cysts with a rigid wall. Origin could not be identified	No evidence of a malignancy, and the cyst was diagnosed by staining as a peritoneal inclusion cyst
Giant ovarian cyst (serous cystadenoma) (Dhuliya et al.) [16]	28 yrs/8 w.o.g. Generalized abdominal distension f/b generalized abdominal pain over 5 months associated with nausea, vomiting, and breathlessness	Findings: huge central abdominal cyst (no solid elements or septations) occupying the entire abdomen and pelvis. Origin could be defined. DDx: non-malignant morphology	Patient is claustrophobic -> MRI not done	Findings: cyst observed - no septations, growth, or masses. Appeared to originate from the left paraovarian region, from the broad ligament, and adherent to the left ovary	The cyst wall is lined by cuboidal epithelium and features consistent with benign serous cystadenoma
Mesenteric cyst (Regupathi and Janakiraman) [17]	18 yrs/12 w.o.g. Diffuse abdominal pain of 1 month's duration	Findings: large abdomino-pelvic mass (multiple septations) of size 22 x 19 x 8 cms. DDx: not mentioned	Findings: not described. DDx: Rt complex ovarian cyst? Mesenteric cyst	Findings: cyst (multiloculated) originating from bowel wall. 2 cysts measuring 10 x 10 with intervening small bowel	Cyst section showed cuboidal epithelium with supporting fibrous tissue, suggestive of a simple mesothelial mesenteric cyst
Retroperitoneal mucinous cyst (Wen et al.) [18]	33 yrs/38 w.o.g. No clinical features mentioned	Findings: cyst (anechoic, no light band separation, no septations, no growths) on the right side of the abdomen of size 12.7 x 14.3 x 24.5 cm. DDx: not mentioned	Findings: cyst (clear borders, homogenous signal) exerting pressure on adjacent structures close to the right ovary, measuring 14.1 x 12.1 x 20.3 cm. DDx: not mentioned	Findings: a large cyst was identified behind the right peritoneum. Cyst extended from the liver margin to the right ovarian pelvic infundibulopelvic ligament of size 13 x 15 x 25 cm	HPE suggested a benign cyst with calcification
Hydatid cyst (Tarafdar et al.) [19]	23 yrs/10 w.o.g. Worsening abdominal pain for 2 days, localized to the RLQ	Findings: multiple cysts in the right adnexa and ovary. Largest: 50 x 60 mm (internal isoechoic septum and elements); another cyst: 81 x 21 x 21 mm (within right adnexa, similar to ovary). Free fluid within the right adnexa, rectouterine pouch, and left adnexa. No detectable blood flow to cysts. DDx: ovarian torsion	Presumed emergency -> MRI not done	Emergency laparotomy attempted in view of ovarian torsion: Findings: cysts - necrotic and attached to the abdominal wall. No origin observed from the ovaries. Cystectomy was done, and the cyst was similar to a hydatid cyst. Numerous other spread over the abdomen and pelvis. Multiple loose adhesions were observed	HPE suggested hydatidosis
Biliary cystadenoma (Raja Navaneethan et al.) [22]	30 yrs/26 w.o.g. Vomiting, abdominal pain, and SOB for 1 day	Findings: anechoic adnexal cyst measuring 13 x 14 x 12.6 cm with multiple thick septations and gross free fluid. PDx: Rt adnexal/ovarian cyst rupture	Presumed emergency -> MRI not done	Purulent ascites with flakes of pus over the duodenum and hepatic flexure of the colon. Thick-walled cysts noted with clear, non-bile-stained fluid arising from under the surface of the liver	

Other presentations that less closely mimic these abdomino-pelvic cysts include appendiceal mucoceles and pancreatic pseudocysts [20,21]. While each author recommends incorporating the specific differential discovered in their case into an extensive list of possibilities for abdomino-pelvic cysts in pregnancy, further studies should devise and validate a diagnostic system that more effectively distinguishes between these. This can facilitate clear and rapid clinical management and avoid the pitfalls faced by the RMFK, as in our case.

The difficult management plan

Tarafdar et al. implemented a similar management approach, in which a case involving multiple hydatid cysts was taken urgently to the operating room (OR) due to abdominal pain suspected to be caused by adnexal torsion [19]. Likewise, Raja et al. have reported an abdomino-pelvic cyst in pregnancy that was interpreted to be of ovarian origin on ultrasound, and a provisional diagnosis of ovarian torsion /rupture was made [22]. The patient was taken urgently to the operating room due to hemodynamic instability. Intraoperatively, after adnexal pathology was excluded, the surgical team was consulted, indicating that the primary surgeon was likely from the OBGYN team, with the general surgery or hepatobiliary team brought in subsequently. They were unexpectedly confronted with a cyst of hepatobiliary origin, but were able to manage the intraoperative finding without causing any harm. This parallels our patient’s experience in the RMFK, where a similar plan was made, but the laparotomy was abandoned, quite possibly due to the lack of specialized surgical expertise at the time, though not without exposing the patient to operative risks.

We are now faced with two key challenges. The first is the influence of clinical framing and urgency on diagnosis and management decisions, as illustrated in the cases reported by both Tarafdar et al. and Raja et al., as discussed above. Second, the constraints of low-resource settings, where advanced imaging and specialized surgical skills are often unavailable [7], thereby limiting management options. Although MRI is the modality of choice in such cases, its availability is limited, particularly in rural areas. Even where MRI is accessible, the high cost and prolonged execution time can deter its routine use and may prompt healthcare teams to make hasty clinical decisions regarding referrals and operative planning [8].

Intervention

The treatment of choice for CCs is typically a complete excision with biliary-enteric reconstruction due to the risk of malignancy [1]. However, laparotomy during pregnancy carries significant maternal and fetal risks - higher incidence of preterm birth, low birth weight, gestational hypertension, and increased rates of cesarean section (CS) [5]. Hence, most CCs are operated on during the first trimester. Also, radical cyst excision surgery during the second and third trimesters is associated with a high risk of postoperative complications, such as bile leakage, and is generally not preferred [23]. Nevertheless, if there are pressing surgical/obstetric indications, the procedure should not be delayed. PCD can effectively alleviate compressive symptoms, cholangitis and also reduce surgical complexity [24]. It also markedly improves imaging anatomy for diagnostic precision, and the reduced risk of complications allows for appropriate intrauterine fetal development [1].

We opted to proceed with PCD under USG guidance into the cyst, as the mother was showing early signs of decompensation (cholangitis, worsening compressive features) and we wanted to avoid an emergent preterm delivery. About 1100 ml of bilious-appearing fluid was aspirated. Cytology was not performed at the time. The procedure dramatically reduced the size of the cyst and relieved pressure. The patient’s fever and tachycardia resolved, and oxygenation improved. Similar positive outcomes have been reported with USG-guided biliary drainage in both antenatal and post-partum cases [4,8].

Outcomes

Total serum bilirubin reached a peak on 31/12, and post percutaneous aspiration (2/1/24), it dropped to 1.83. The other parameters also followed suit, showing marked improvement post percutaneous aspiration. ALP and GGT (standard markers of cholestasis) showed a gradual drop over the coming week. Serial monitoring of the pigtail drain showed a decreasing output (1600 -> 600 -> 400 -> 350 ml). On PPD-4, the patient had three episodes of bilious vomiting, indicating unrelieved obstruction. A multidisciplinary team discussion was held with the patient and attenders. The condition and associated maternal and fetal risks were explained. Management options and their respective outcomes and risks were provided: (1) continuation of the pigtail catheter until fetal maturity; (2) surgical cyst resection; or (3) referral to a higher center. The patient and attendants opted to continue treatment and consented to surgery at SDM hospital. 

In uncomplicated cases, conservative management until delivery, followed by cyst excision after planned vaginal delivery or elective cesarean, remains the standard approach, reflecting the 'dual-step' strategy described by Jia et al. [25]. Augustine et al. also support this two-stage approach for biliary complications in the third trimester, starting with ultrasound-guided drainage (unless there is fetal distress), and proceeding to surgical intervention if symptoms are not adequately managed [1]. An exploratory laparotomy + excision of CC + hepatojejunostomy + jejunojejunostomy was undertaken. OBG and the pediatric team were informed beforehand to ensure intraoperative support if needed. Surgical exploration confirmed the presence of a CC. Biopsy confirmed the diagnosis of CC with spongioid hyperplasia of the gall bladder. Postoperatively, the patient was managed with IV antibiotics, analgesia, and anti-inflammatory medication. 

The optimal mode of delivery in pregnancies complicated by a CC remains a subject of debate. Some authors advocate for elective cesarean delivery to avoid the physical stress of labor and the theoretical risk of cyst rupture [7]. In contrast, a systematic review found no reported cases of CC rupture during either spontaneous or induced vaginal delivery, suggesting that CS should be reserved for specific indications such as fetal distress, surgical emergencies, progressive cholangitis, or symptoms that do not respond to conservative management. In our case, both temporizing and definitive surgical intervention had already been undertaken, obviating the need for elective CS and permitting a favorable obstetric environment for normal vaginal delivery.

Recommendations

**Figure 12:**
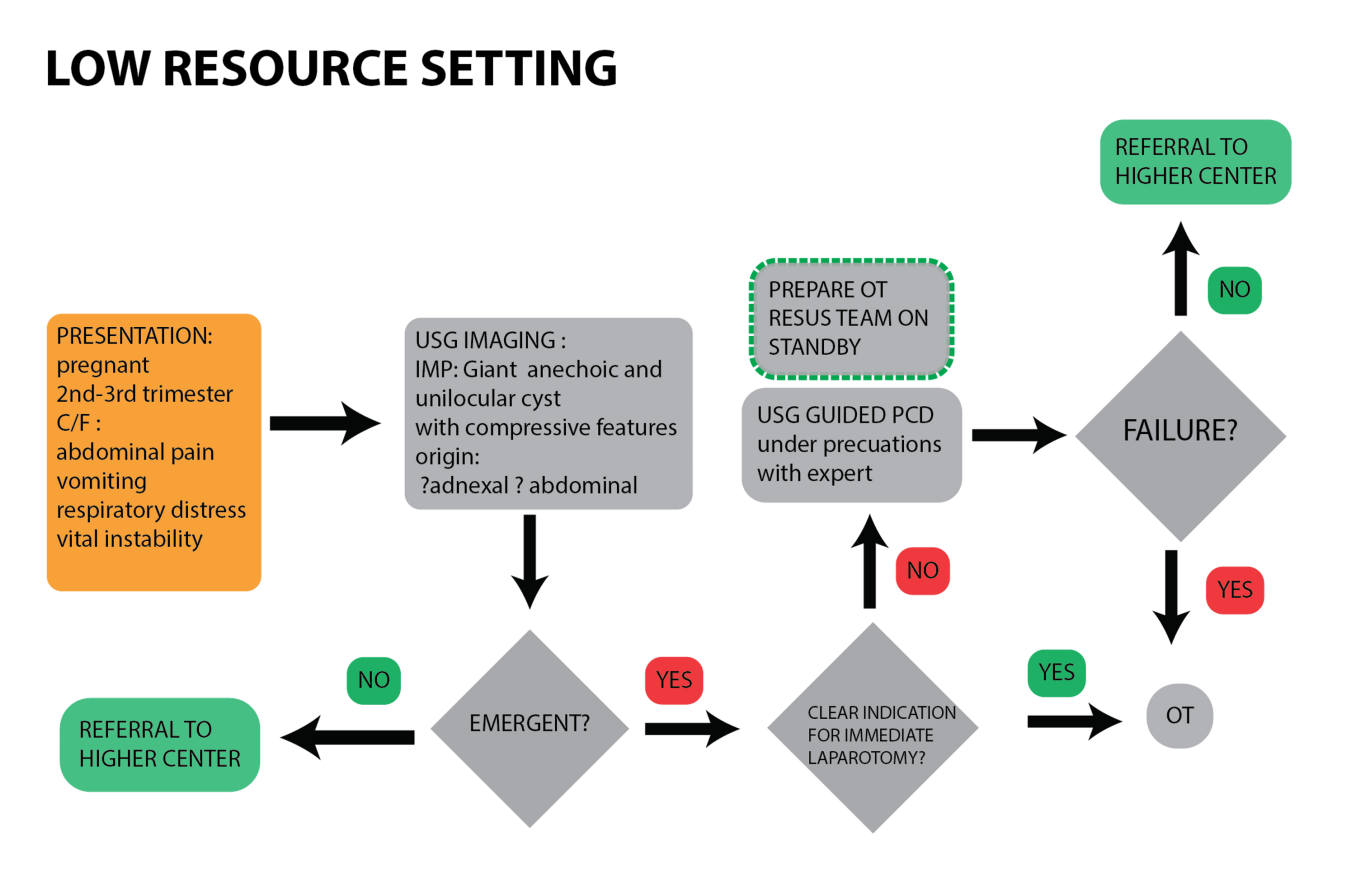
Application of USG-PCD as an emergency management in pregnant patients with large symptomatic abdomino-pelvic cysts in rural settings USG: ultrasound; PCD: percutaneous catheter drainage; C/F: clinical features, IMP: impression; OT: operation theater

In pregnant patients with a large abdominal or adnexal cyst, ultrasound can confirm the presence of a cystic lesion but may fall short in clearly defining the anatomy needed for an accurate diagnosis. This limitation becomes particularly significant when the suspected condition is an emergency with a limited window for intervention, such as ovarian torsion or a ruptured ovarian cyst. In such situations, the typical response in many clinical settings is to proceed directly to emergency laparotomy. While this may be relatively safer in non-pregnant patients, it poses considerable risks to both mother and fetus during pregnancy [5]. Below, we outline a hypothetical strategy aimed at reducing maternal and fetal morbidity and mortality in cases of large abdominal or adnexal cysts presenting with symptoms during pregnancy (Figure 12). For brevity, we refer to this as the "needle before knife" approach.

Emergency USG-Guided PCD (USG-PCD)

**Figure 13:**
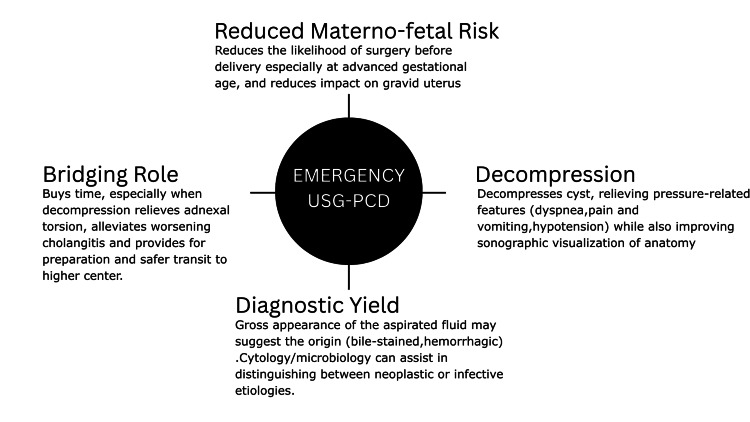
Advantages of emergency USG-PCD for large symptomatic simple abdomino-pelvic cysts during pregnancy

In resource-limited settings where MRI or MRCP is not readily accessible, we propose that ultrasound-guided percutaneous drainage (USG-PCD) can be used as a practical interim solution following the initial ultrasound screening. This approach offers four important advantages: decompression, diagnostic Yield, bridging role, and reduced maternal-fetal risk (Figure 13).

The method is not a ‘one-size-fits-all' approach, and only applies to this very specific presentation when resources are not available. Potential objections to this approach may arise in cases involving ovarian torsion, ruptured ovarian cysts, or hydatid cysts. 

Adnexal Torsion and Adnexal Malignancy

Traditionally, laparotomy has been the standard approach for managing adnexal torsion and other acute cystic conditions. However, recent evidence supports a less invasive stepwise approach. Berg et al. analyzed 46 cases of adnexal torsion and demonstrated that ultrasound-guided cyst aspiration achieved complete resolution in 85% of cases, with an even higher success rate in pregnant patients (92%). To exclude malignancy, the study employed the pattern recognition method performed by Level 3 ultrasonographers for differentiating benign from malignant lesions. [26]

Based on their findings, the authors recommend aspiration (transabdominal or transvaginal) for unilocular cysts containing anechoic or hypoechoic fluid as a treatment for adnexal torsion, effectively eliminating surgical risk. Also, significantly, post-procedure cytology in all cases revealed no evidence of malignancy. The underlying mechanism is straightforward: complete aspiration of the cyst relieves the torsional force applied to the pedicle. Clinically, torsion is often suspected based on symptoms, and pregnancy-related anatomic changes can increase the likelihood of misidentifying non-gynecological cysts as adnexal emergencies. Analyzing which cysts are suitable for aspiration or ultrasonography is critical, as timely intervention can alleviate pressure-related symptoms and reduce the risk of torsion.

Ruptured Ovarian Cysts

While surgery may be required for hemodynamic instability or torsion, most ruptures are self-limiting. Kim et al. have reported that 80% of women with hemoperitoneum were successfully managed conservatively with USG-PCD [27]. Surgical intervention was rarely needed in stable patients without large-volume hemoperitoneum or low diastolic blood pressure (DBP) (10%).

Hydatid Cysts 

Hydatid disease poses the unique risk of anaphylaxis upon rupture. Though classic ultrasound signs (e.g.,”cyst-within-cyst,” “water lily”) aid in its recognition, some lesions mimic simple anechoic, unilocular cysts. In cases of uncertainty, the cyst should be treated as hydatid until proven otherwise, and careful ultrasound-guided drainage using the PAIR (Puncture, Aspiration, Instillation, and Reaspiration) technique, performed by an experienced specialist with proper precautions and antibiotic coverage, may be considered [28].

Limitations and challenges**

In Berg et al.'s study, the diagnosticians were highly skilled sonographers tasked with identifying torsion, distinguishing benign from malignant cysts, and performing USG-guided PCD. However, in resource-limited settings, both the availability of such expertise and the quality of sonographic equipment may be limited, posing a significant challenge. While non-malignant cysts often appear unilocular and anechoic or hypoechoic, it cannot be assumed that 100% of all other non-adnexal cysts (abdominal) with these features are benign, requiring a more comprehensive evaluation.

Importantly, the dilemma extends beyond the risks of puncture alone. Large intra-abdominal cysts can themselves compromise patient stability, causing respiratory distress from diaphragmatic splinting, desaturation, tachypnea, hypotension from vascular compression, or even rupture. In some cases, complications such as cholangitis may develop depending on the cyst's nature and location. Thus, clinicians must balance the potential hazards of intervention against the equally serious consequences of non-intervention in a symptomatic patient. By applying meticulous precautions and careful case selection, the approach aims to minimize risk while addressing a clinical problem that, if left unattended, may pose even greater harm.

MRI-MRCP continues to be the diagnostic gold standard; however, when it is not available, a "needle-before-knife" approach, prioritizing ultrasound-guided aspiration, can provide a safe and potentially life-saving alternative. It should be noted that this novel approach is conceptual, not protocolized. Its safety and feasibility warrant systemic evaluation within a multidisciplinary framework. If validated, it could tremendously reduce unnecessary laparotomies and improve maternal-fetal outcomes in resource-limited settings.

## Conclusions

This case illustrates how initial misdiagnosis of a CC in pregnancy can lead to unnecessary laparotomy, with significant maternal and fetal risks. Advanced imaging techniques, especially MRI and MRCP, play a crucial role in achieving an accurate diagnosis and should be used whenever accessible. MRCP, in particular, is regarded as the gold standard for visualizing the biliary tree during pregnancy without exposing the patient to ionizing radiation. In settings where MRI/MRCP access is limited, careful ultrasound assessment with an experienced operator remains essential, although gravid anatomy may obscure critical findings. Timely percutaneous drainage can serve as a safe bridge to definitive surgery, relieving mass effect and reducing the risk of complications such as rupture, cholangitis, or pancreatitis. Materno-fetal outcomes are optimized when a multidisciplinary approach - combining radiology, surgery, and obstetrics - is adopted. In resource-limited environments where a symptomatic large abdominal/adnexal cyst is discovered in pregnancy, and a based diagnosis is inconclusive, adopting a 'needle before knife' approach - performing USG-guided PCD before rushing to laparotomy - in select cases, can reduce morbidity and mortality while also improving maternal-fetal outcomes.
